# Analysis of research hotspots in COVID-19 genomics based on citespace software: Bibliometric analysis

**DOI:** 10.3389/fcimb.2022.1060031

**Published:** 2022-12-12

**Authors:** Xue meng Pang, Zhao yun Peng, Xin Zheng, Jing jing Shi, Bao chen Zhou

**Affiliations:** ^1^ Shandong University of Traditional Chinese Medicine, Jinan, Shandong, China; ^2^ The Second Affiliated Hospital of Shandong University of Traditional Chinese Medicine, Jinan, Shandong, China; ^3^ Qingdao Hospital of Traditional Chinese Medicine (Qingdao Hiser Hospital), Qingdao, Shandong, China

**Keywords:** bibliometric analysis, visual analysis, research hotspots, CiteSpace software, genomics, COVID-19

## Abstract

**Introduction:**

To analyze the current state, hotspots, and cutting-edge trends of genomics research on the outbreak of Corona Virus Disease 2019 (COVID-19) from 2019 to the present (March 2022).

**Methods:**

Statistical and visual analysis of COVID-19 genomics results published in the 2019-2022 Web of Science Core Collection Database (WOSCC) was performed using CiteSpace software, including data on countries, institutions, authors, journals, co-citations, keywords, etc.

**Results:**

A total of 9133 English literature were included. The number of publications has significantly increased in 2021, and it is expected that this upward trend will last into the future. The research hotspots of COVID-19 revolve around quarantine, biological management, angiotensin-converting enzyme-2, RNA-dependent RNA polymerase, etc. Research frontiers and trends focus on molecular docking, messenger RNA, functional receptor, etc.

**Conclusion:**

The last two years have seen a significant increase in research interest in the field of novel coronavirus pneumonia genomics.

## Introduction

The Corona Virus Disease 2019 (COVID-19), caused by Severe Acute Respiratory Syndrome Coronavirus 2 (SARS-CoV-2), has resulted in millions of confirmed cases and fatalities worldwide (Safiabadi et al., 2021; Zhong and Lin, 2022) and has led to a global crisis that has elevated to the status of a public health emergency global concern (Mughees et al., 2021). The most common clinical symptoms of COVID-19 disease are respiratory symptoms, such as dry cough, fever, and shortness of breath. Some patients also experience other symptoms, including abdominal pain and diarrhea, neurological damage, headache, myalgia, and fatigue (Chen et al., 2020). A recent study of 401 cases (aged 51-81 years) who tested positive for SARS-CoV-2 infection found greater reductions in overall brain volume and greater cognitive deterioration between time points (Douaud et al., 2022). Antiviral drugs currently commonly used to treat ribavirin (nucleoside analogs), lopinavir/ritonavir (protease inhibitors), remdesivir and favipiravir may be potentially effective in the treatment of COVID-19, including IL-6 monoclonal antibodies, tyrosine-protein kinase 1/2 inhibitors, cell therapy drugs, etc (Savarino et al., 2006; Yan et al., 2013; Nie and He, 2020; Seyed et al., 2020).

The genomic study of the new coronary pneumonia epidemic has gained attention due to potential mutations in the internal gene sequences of the SARS-CoV-2 since the Human Genome Project’s completion and the advent of bioinformatics technology. The knowledge based on the impact of SARS-CoV-2 spike mutations on antigenicity and other aspects of viral biology is rapidly expanding, and the integration of gene sequences has the potential to facilitate potentially interesting variants (Harvey et al., 2021). Global genomics-based monitoring of new variants of COVID-19 will continue to be a key component in collecting and making available online information on all SARS CoV-2 lineages, enabling rapid assessment of their influence on transmission, virulence, and vaccine escape (Knyazev et al., 2022). Understanding the structural and functional characteristics of SARS CoV-2 protein is crucial to promote the development of anti-CoV drugs and vaccines that could prevent and control the current SARS CoV-2 pandemic (Bai et al., 2022). Sali Abubaker Bagabir et al. used artificial intelligence technology (AI) to open the door to the genome sequence of the COVID-19 virus and variants of concern (VOC), and found that spike protein was the gene with the most genes among genes for vaccine discovery (Abubaker et al., 2022).

Bibliometric analysis is a popular method for analyzing large amounts of scientific data. It discovers knowledge associations between documents by filtering and processing large amounts of information and is used in various applications such as biomedicine, economic management, information science, renewable energy, and the environment (Yu et al., 2018; Hassan et al., 2021; Zhang et al., 2022). The use of bibliometric theories and methods to scientifically analyze specific journals is one of the important topics of bibliometrics (Zhang and Lin, 2022), which provides a comprehensive perspective for conducting research and is of great significance for scholars to grasp the hot spots in this field promptly (Chen et al., 2022). CiteSpace is designed by Professor Chen for quantitative information acquisition and visualization in specific fields and is considered to be one of the most influential software in the field of bibliometrics and information visualization (Lin et al., 2021; Shen et al., 2022).

This study used CiteSpace software (version 5.6. R3) to visualize and analyze the pertinent literature on novel coronavirus pneumonia genomics published in the WOSCC Database from 2019 to 2022, and to summarize the research status and hot frontiers of COVID-19 in the field of genomics, which provides reference and ideas for the development of COVID-19 genomics targeted laboratory characterization studies and the updating of antigen variant vaccines.

## Materials and methods

### Data sources

Search strategy: TI= (“COVID 19” OR “atherosclerotic” OR “Severe Acute Respiratory Syndrome Coronavirus 2 Infection” OR “SARS-CoV-2” OR “Coronavirus Disease 19” OR “Coronavirus Disease 2019” OR “2019 Novel Coronavirus*”) AND TS= (“genomics” OR “genome*” OR “DNA” OR “RNA”). Time span: 1999~2022; Index: SCI-EXPAND-ED. The types of included documents are “Article” and “Review”, and the language is “English”.

### Method

CiteSpace software (Yu et al., 2019) was used to visualize and analyze COVID-19 genomics-related literature in the WOSCC. The SCI-EXPANDED WOSCC Database was selected because it efficiently represented the search results. Therefore, it is recommended to use it to search for journals and references (Usman and Ho, 2021). The specific method is to use CiteSpace software to integrate and deduplicate, finally obtain a total of 9133 documents, analyze the data of countries, institutions, authors, journals, co-cited references, keywords, and other data and draw a visual map. The software time parameters are set from 2019 to 2022 with a time slice of 1 year. The appropriate thresholds were selected according to different nodes, and the map clipping methods were set to Pathfinder, Pruning sliced networks, and Pruning the merged network.

## Results

### Literature distribution

**Figure 1:**
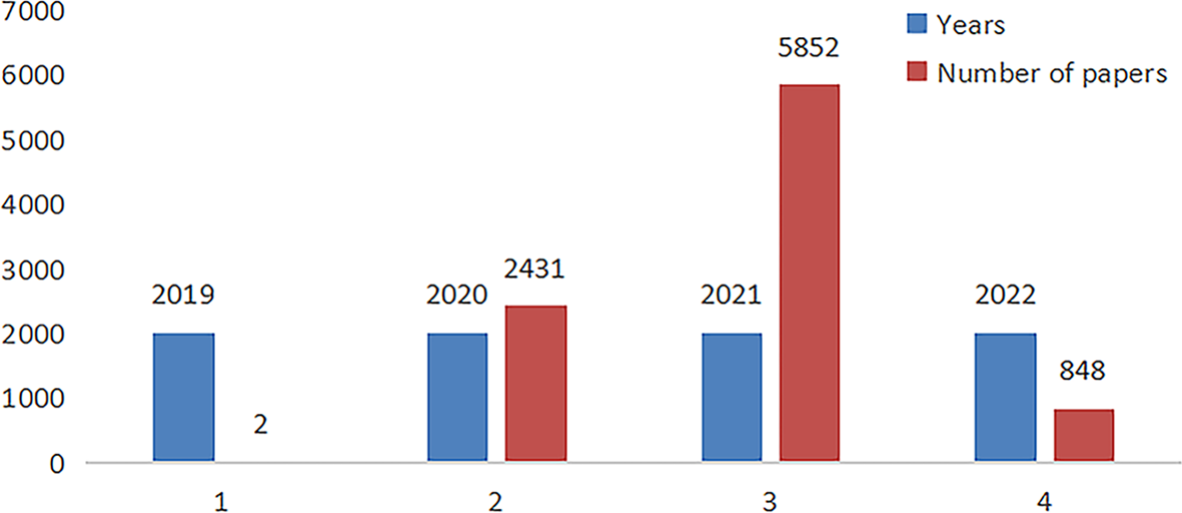
Distribution of papers in COVID-19 genomics from 2019 to 2022.

A total of 9133 articles were retrieved and exported in plain text format from 2019 to 2022. In 2019, two papers will be published, which is the outbreak period of COVID-19; 2,431 papers will be published in 2020, which is a growth period; 5,852 papers will be published in 2021, which is a period of rapid growth and research hotspot; 848 papers will be published in March 2022, and a continuous upward phase. Research related to genomics is ongoing with the outbreak of COVID-19 and the growing awareness of research needs and potential. The research field of COVID-19 is very diverse and is actively explored by experts and scholars in different disciplines around the world (**Figure 1**).

### Co-occurrence analysis of countries and institutions

**Figure 2:**
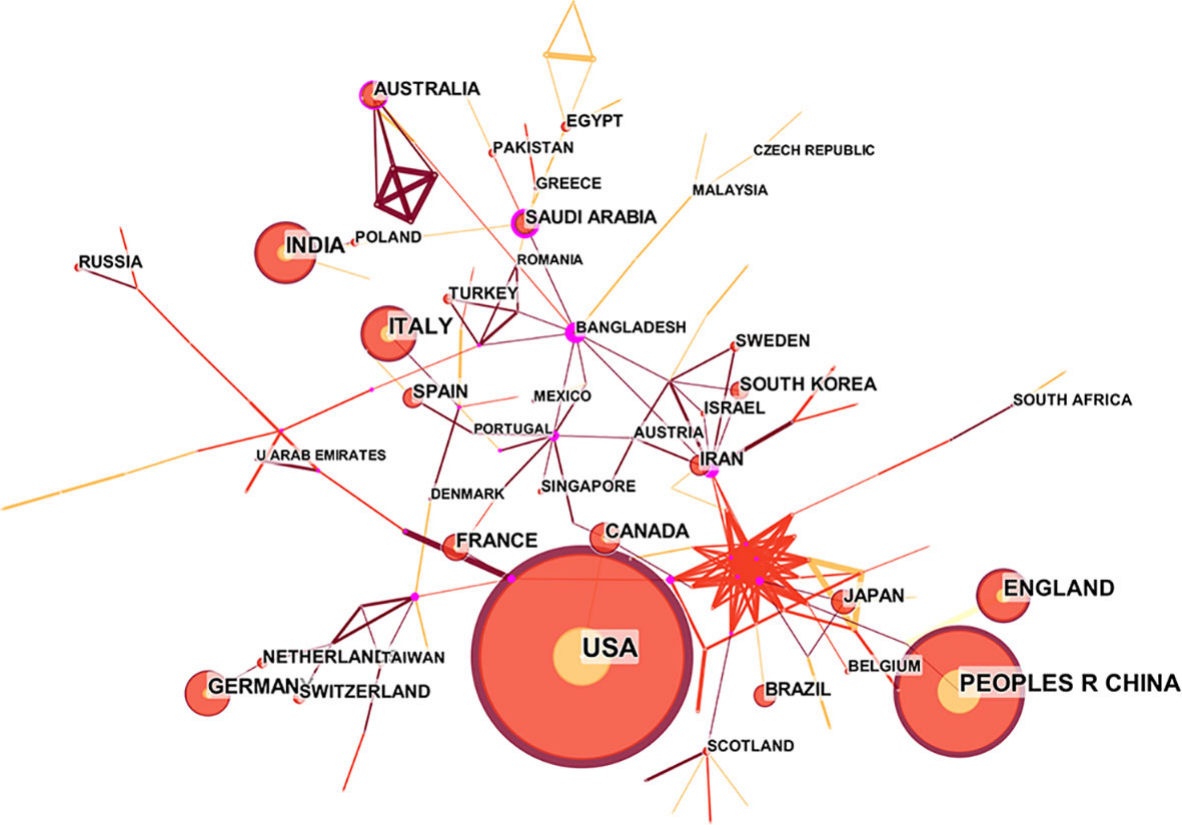
National collaboration map for COVID-19 genomics research.

**Figure 3:**
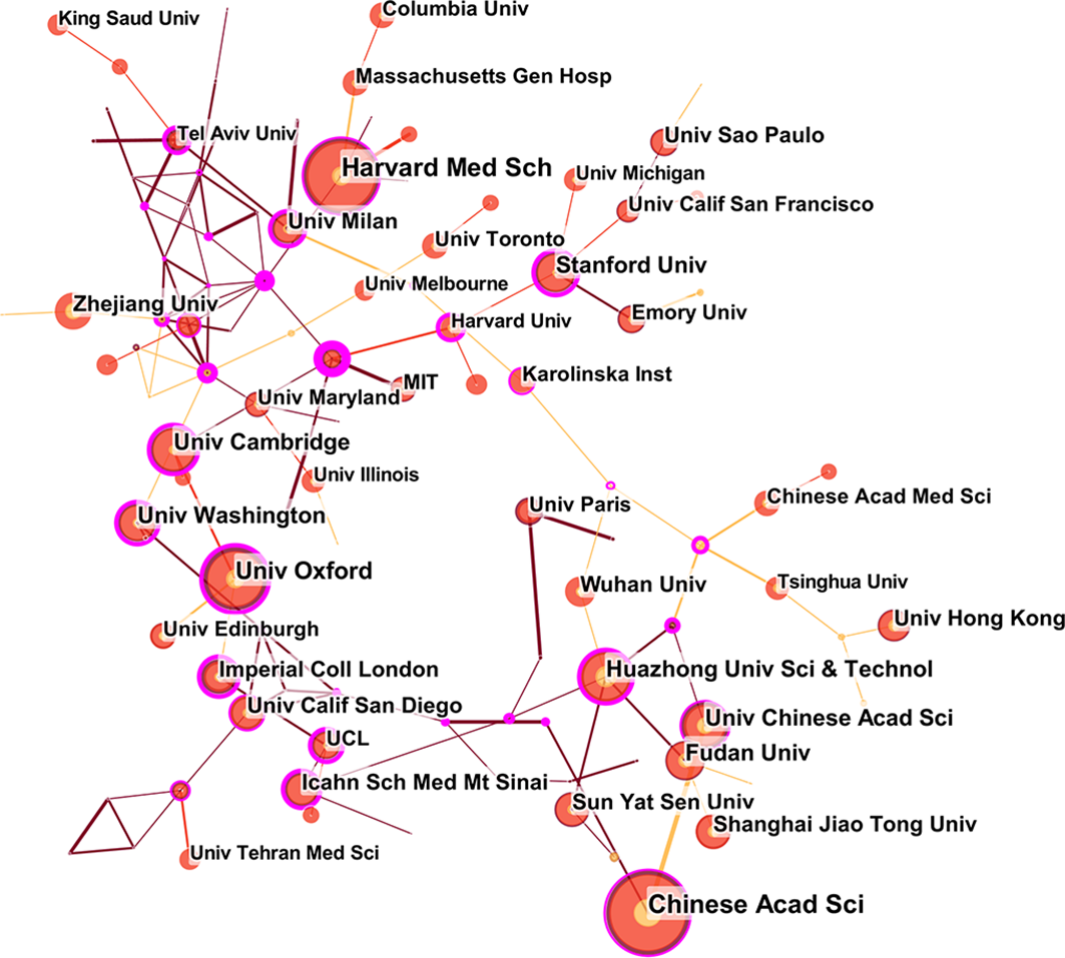
Institutions collaboration map for COVID-19 genomics research.

The 50 Countries and Institutions with the highest number of occurrences each year are selected for data statistics and visual analysis. Each node represents a country or institution and the connection between nodes represents the cooperation between countries or regions (Yu et al., 2020). **Figure 2** shows the cooperative network publications between at least 10 countries or regions. Through this diagram, we can find that the number of papers published by the United States and China is significantly higher than that of other countries, but the centrality is 0, indicating that the United States and China are the global leaders in this field, but have less international cooperation. The top 10 institutions with the most published papers are American universities and related medical institutions in China, suggesting that Chinese Acad Sci and Harvard Med Sch have more in-depth research on COVID-19 genomics. This paper provides evidence to support the importance of genomics in COVID-19 and provides some rationale for it. It is worth noting that, as seen in **Table 1**, most of the countries with the highest productivity of COve number of articles is closely related to economic development (**Figures 2**, **3**, **Table 1**).

**Table 1:** Top 10 countries and institutions with the number of publications on COVID-19 genomics.

Ranking	Country			Institution		
	Country	Frequency	Centrality	Institution	Frequency	Centrality
1	USA	2680	0,00	Chinese Acad Sci	186	0.00
2	CHINA	1620	0.00	Harvard Med Sch	168	0.14
3	INDIAN	775	0,04	Univ Oxford	138	0.15
4	ITALY	730	0.00	Huazhong Univ Sci & Technol	111	0.17
5	ENGLAND	695	0.00	Univ Chinese Acad Sci	106	0.19
6	GERMANY	603	0.00	Univ Cambridge	97	0.23
7	CANADA	388	0.03	Stanford Univ	94	0.00
8	FRANCE	361	0.00	Fudan Univ	93	0.00
9	AUSTRALIA	334	0.12	Univ Washington	92	0.32
10	JAPAN	327	0.02	Imperial Coll London	88	0.16

### Analysis of author co-occurrence and co-citation

#### Author co-occurrence

The 30 authors with the highest number of occurrences each year were selected for co-occurrence analysis and co-citation analysis. To prevent the problem of homonymy among different authors, the authors with the top 10 published papers were included in this study for manual screening and frequency statistics after download. The results indicated that the authors with higher publication volume had more in-depth research on COVID-19 genomics but with lower centrality, suggesting a low level of cooperation between authors; 19 Research and development in the field of genomics is of great significance.

**Figure 4:**
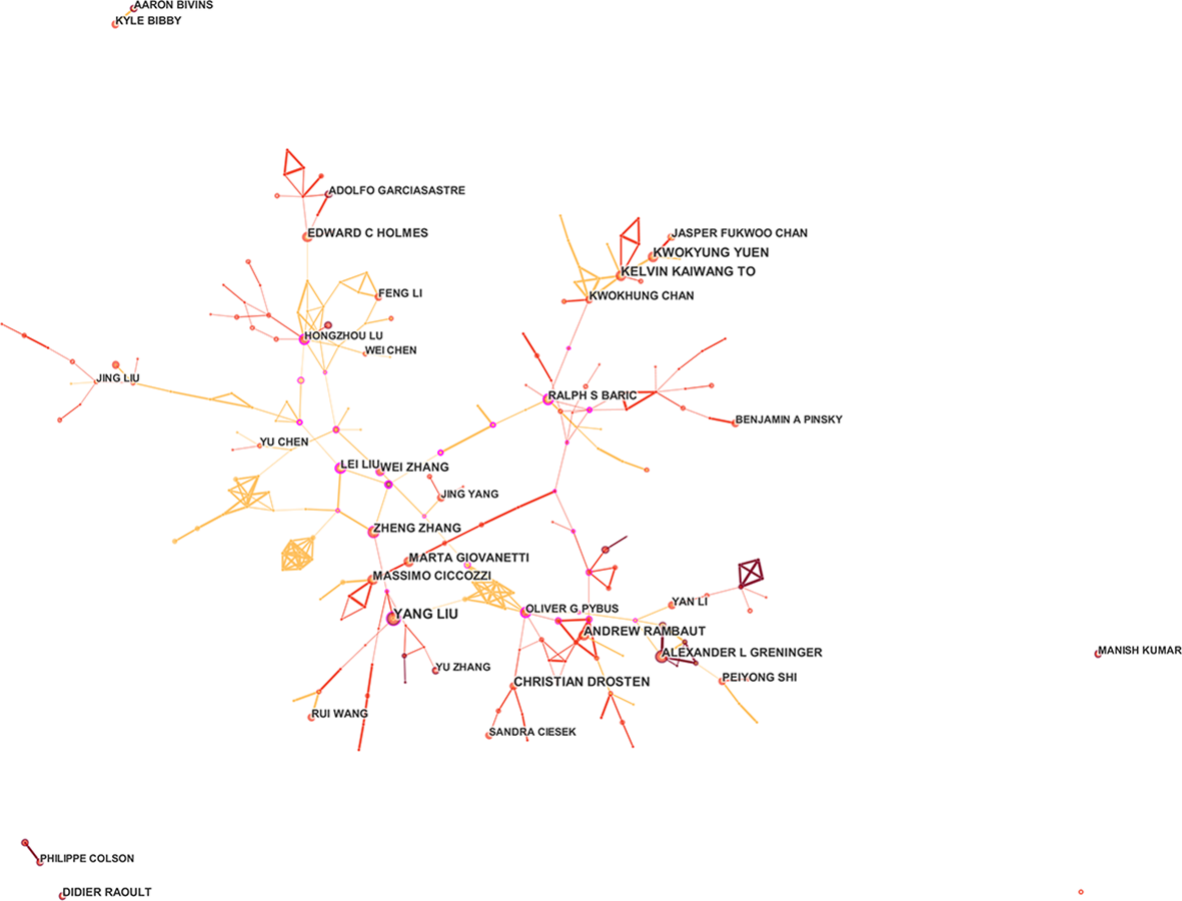
Author co-occurrence map of COVID-19 genomics.

YANG LIU is the most productive author in terms of the number of publications. They mainly focus on the COVID-19 single-cell transcriptome atlas and the immune characteristics of single-cell sequencing (Zhu et al., 2020; Ren et al., 2021). CHRISTIAN DROSTEN, KWOKYUNG YUEN, KELVIN KAIWANG TO, ANDREW RAMBAUT, and others are also active in this field. From the network map (**Figure 4**), we can find close annotation among the top 10 authors, which identifies the close collaboration of these professional authors, and most of the research results are from teams specializing in the field of treating COVID-19 genomics.

#### Author co-citation

**Figure 5:**
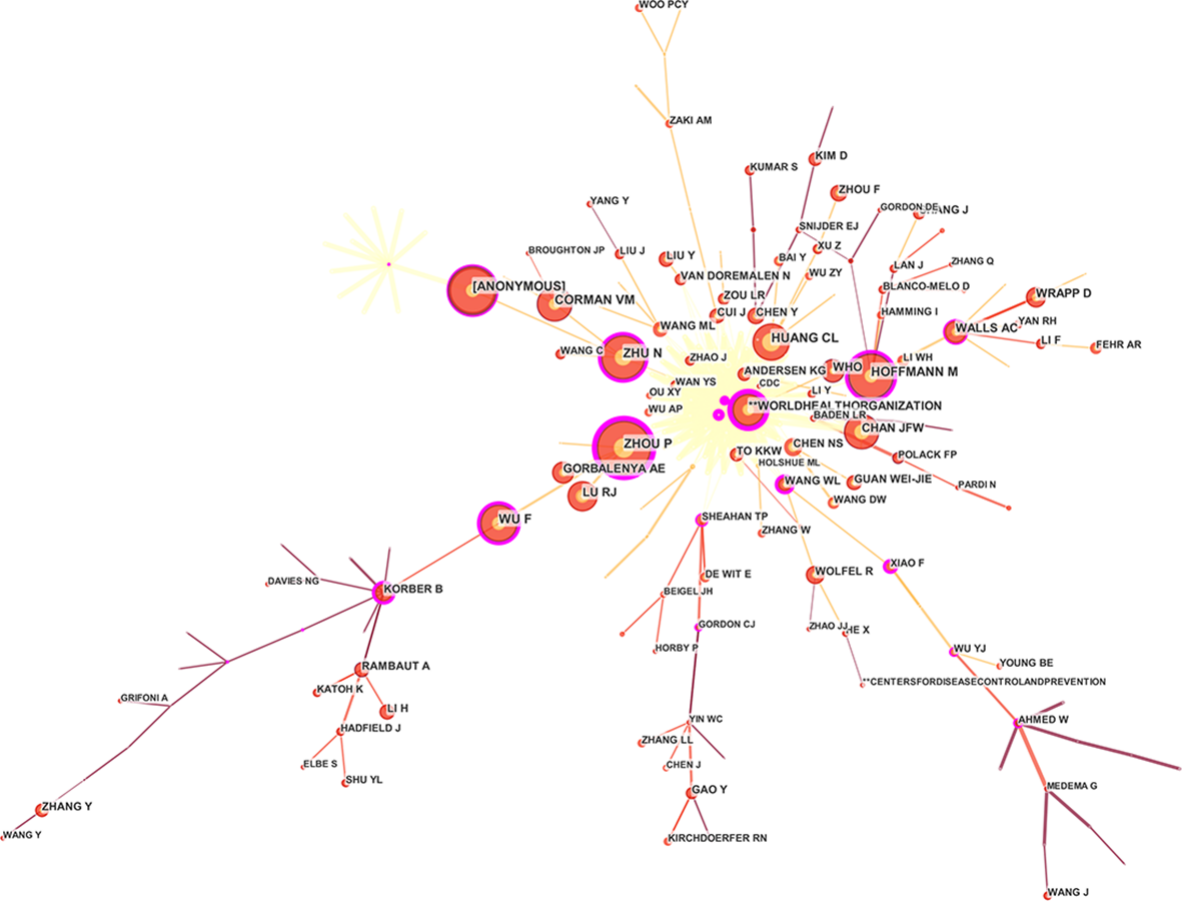
Author co-citation map of COVID-19 genomics.

Co-citation refers to the phenomenon that two or more authors or their articles are cited by other literature at the same time (Pan et al., 2021). Among the author co-citation, the most cited author was Zhou P, with citation counts of 1694, followed by Anonymous, Zhu N, Hoffmann M, and Huang CL (**Figure 5**, **Table 2**). From the cluster review, the authors devoted their minds to Sars-Cov-2, vaccines, and angiotensin-converting enzyme 2 (ACE2) (Jiang et al., 2020), as the keywords ranked in the top five.

**Table 2:** Top 10 authors and co-cited authors in research of COVID-19 genomics.

Ranking	Author’s publication volume			Co-cited author		
	Author	Frequency	Centrality	Author	Frequency	Centrality
1	YANG LIU	32	0.11	Zhou P	1694	0.40
2	CHRISTIAN DROSTEN	25	0.03	Anonymous	1528	0.17
3	KWOKYUNG YUEN	25	0.01	Zhu N	1411	0.25
4	KELVIN KAIWANG TO	23	0.06	Hoffmann M	1398	0.28
5	ANDREW RAMBAUT	21	0.01	Huang CL	1222	0.08
6	MASSIMO CICCOZZI	20	0.03	Wu F	1182	0.34
7	WEI ZHANG	20	0.12	Corman VM	1157	0.03
8	MARTA GIOVANETTI	20	0.04	Chan JFW	1092	0.02
9	EDWARD C HOLMES	20	0.06	**World Health Organization	998	0.58
10	ZHENG ZHANG	20	0.14	Lu RJ	965	0.00

**means World Health Organization (WHO), WHO is a specialized agency under the United Nations and the largest intergovernmental health organization in the world.

#### Analysis of journal co-citation and double graph overlay

**Figure 6:**
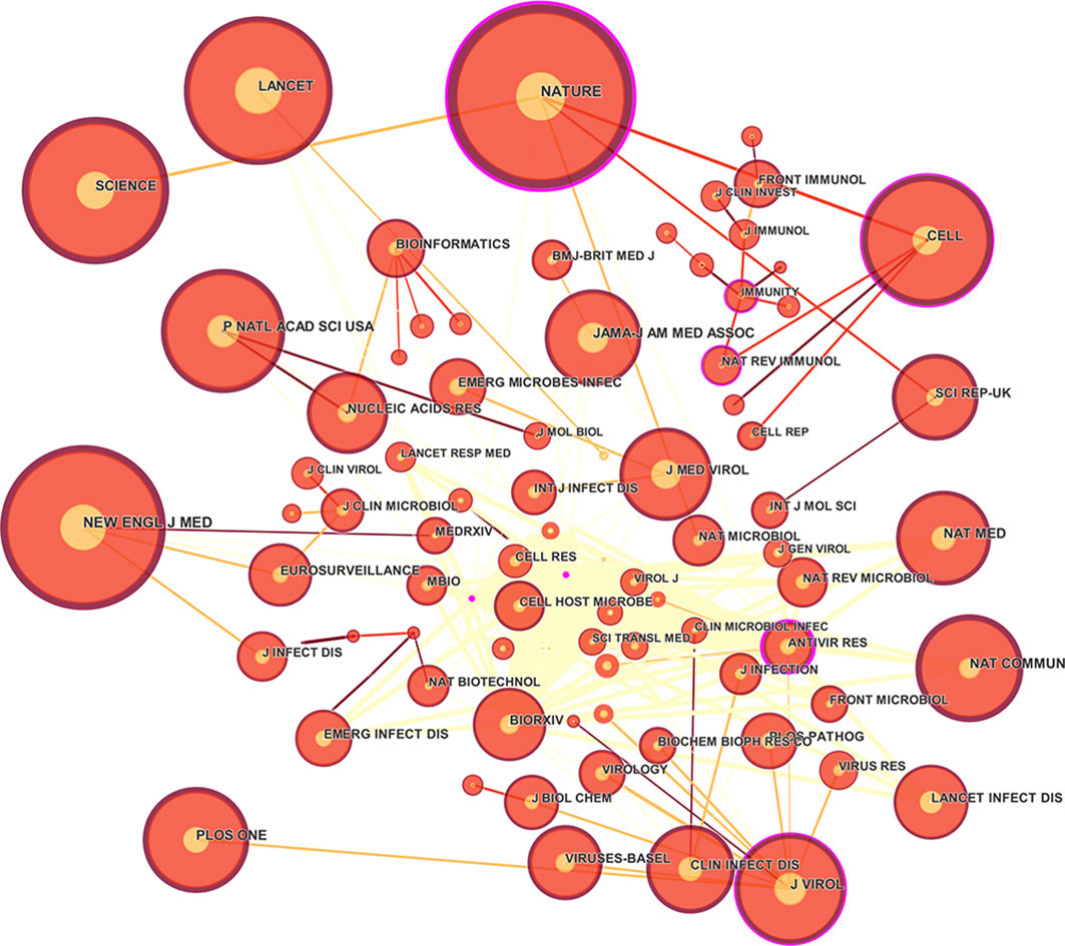
Journal co-citation map of COVID-19 genomics.

The 30 most cited journals each year were selected for visual analysis. The results showed that the top 10 most cited journals had higher impact factors. Among them, there are 8 journals with an impact factor greater than 10, namely NEW ENGL J MED, LANCET, JAMA-J AM MED ASSOC, NATURE, SCIENCE, CELL, NAT COMMUN, P NATL ACAD SCI USA, with the highest impact factor of 90.333, indicating that the research in the field of COVID-19 genomics has high research value in the field of medical genomics (**Figure 6**, **Table 3**).

**Table 3:** Top 10 journals cited in research of atherosclerosis genomics.

Ranking	Journal	Frequency	Centrality	IF Impact factor
1	NATURE	5711	0.25	49.462
2	NEW ENGL J MED	5140	0.10	90.333
3	LANCET	4663	0.02	78.528
4	SCIENCE	4527	0.00	47.728
5	CELL	4090	0.18	41.582
6	P NATL ACAD SCI USA	3890	0.10	11.093
7	JOURNAL OF VIROLOGY	3393	0.16	5.052
8	NATURE COMMUNICATION	3358	0.00	14.77
9	PLOS ONE	3330	0.00	3.208
10	JAMA-J AM MED ASSOC	3046	0.02	55.709

IF, Impact factor; NEW ENGL J MED, New England Journal of Medicine; P NATL ACAD SCI USA, Proceedings of the National Academy of Sciences of the United States of America; JAMA-J AM MED ASSOC, Jama-Journal of the American Medical Association.

**Figure 7:**
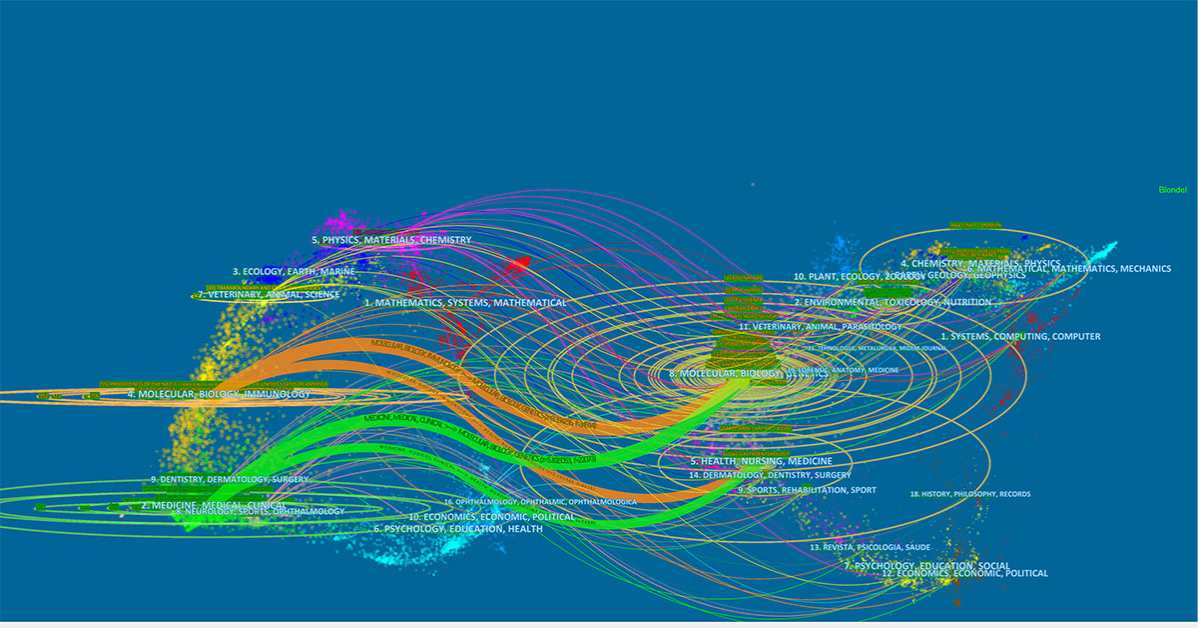
Overlay of dual images of journals of COVID-19 genomics.

The 9133 retrieved literature were imported into the CiteSpace software and a double-map overlay of the journals was plotted. The left side is the subject areas of the cited journals, the right side is the subject areas of the cited journals, and the wavy curve is the citations of the journals. The trajectory, that is, the transfer from the subject area of the cited journals to the subject area of the cited journals, is analyzed through the journal double graph overlay to reflect the knowledge flow between the disciplines involved in COVID-19 genomics literature at the journal level. The results show that the cited journals in the orange citation track are concentrated in molecular, biology, immunology, and other disciplines, and the green ones are concentrated in medicine, pharmacy, clinical and other disciplines. The transfer of disciplines such as COVID-19 science and other disciplines indicates that the disciplines concentrated in the covid-19 genomics-related journals have shifted from the fields of molecular biology, biology, immunology, medicine, pharmacy, and clinical to the fields of molecular biology, genetics, etc. The orange path in **Figure 7** indicates that the literature published in the journal Molecular/Biology/Genetics is frequently cited in the journal Molecular/Biology/Immunology. Therefore, COVID-19 genomics is closely integrated with basic and clinical disciplines, and the construction of multidisciplinary management needs to be further strengthened in the future (**Figure 7**).

### Analysis of keywords

#### Analysis of keyword co-occurrence

**Figure 8:**
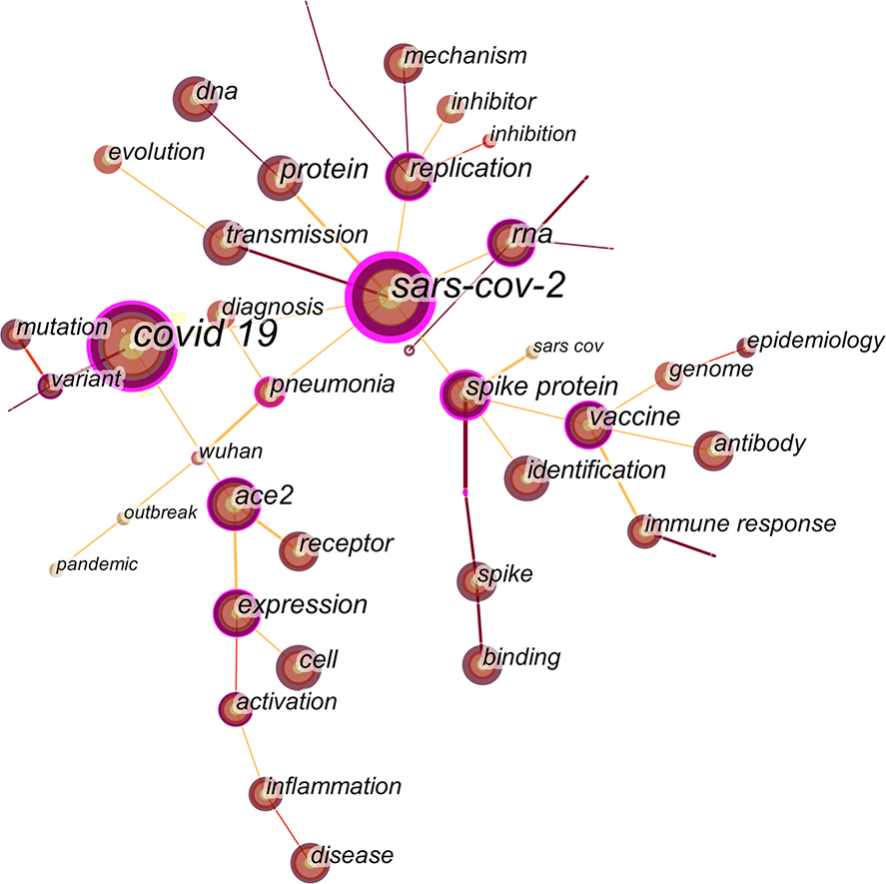
Keywords co-occurrence map of COVID-19 genomics.

By analyzing the frequency and centrality of the keywords, the research frontiers can be identified (Pei et al., 2019). Keywords were manually merged and deleted, such as changing covid-19 to covid 19, and changing sar, sars, virus, coronavirus, infection, respiratory syndrome coronavirus to sars-cov-2. Keywords are high-level summaries of literature content and topics, which could diagnose and activate disease scripts and are an integral part of medical culture and language (Chan and Eppich, 2020). T The results show that research hotspots in the field of COVID-19 genomics mainly focus on covid 19, SRAS-COV-2, protein, RNA, ace2, replication, vaccine, expression, spike protein, transmission, and so on. The studies of COVID-19 genomics are developing rapidly and are now of great research value, playing an important role in the development of biomedicine and human health (**Figure 8**, **Table 4**).

**Table 4:** Top 10 keywords for research in COVID-19 genomics.

Ranking	Keywords		
	Keywords	Frequency	Centrality
1	covid 19	5666	0.84
2	sars-cov-2	3786	1.13
3	protein	570	0.06
4	RNA	518	0.23
5	ACE2	467	0.33
6	replication	406	0.29
7	vaccine	397	0.29
8	expression	377	0.23
9	Spike protein	376	0.57
10	transmission	339	0.06

#### Analysis of keywords bursts

The emerging analysis of keywords reveals the directions and trends in this field’s study during a specific period. After doing a burst keywords analysis on the co-occurrence findings of the keywords, a total of 20 emergent words were obtained. Based on the timing of emergence, these words may be roughly split into three categories: (1) 2019-2020 emergent words are quarantine, biological management, poaching, RNA-dependent RNA, polymerase, drug, bat, hand hygiene, angiotensin-converting enzyme-2, China, conservation, social distancing; (2) 2020-2021 emergent words are wuhan, influenza, functional receptor, pneumonia, sars-cov, pathogenesis; (3) 2021-2022 emergent words are molecular docking, messenger RNA, Wuhan, functional receptor, pneumonia, SARS-COV, and pathogenesis were a few of them, listed in order of their emerging intensity from high to low. Most of the co-cited literature has been frequently cited in the past three years, which implies that the outbreak of studies related to COVID-19 genomics is likely to continue in the future (**Table 5**). As shown in **Table 5**, there have been three periods of keyword bursts since 2019, and COVID-19 genomics research may continue to grow in the future.

**Table 5:** Bursts in research of atherosclerosis genomics.

Keywords burst	Year	Strength	Begin	End	2019-2022
quarantine	2019	0.6924	2019	2019	▃▂▂▂
biological management	2019	0.6924	2019	2019	▃▂▂▂
poaching	2019	0.6924	2019	2019	▃▂▂▂
rna-dependent rna polymerase	2019	0.6924	2019	2019	▃▂▂▂
drug	2019	0.6924	2019	2019	▃▂▂▂
bat	2019	0.6924	2019	2019	▃▂▂▂
hand hygiene	2019	0.6924	2019	2019	▃▂▂▂
angiotensin-converting enzyme-2	2019	0.6924	2019	2019	▃▂▂▂
China	2019	0.6924	2019	2019	▃▂▂▂
wuhan	2019	38.1936	2019	2020	▃▃▂▂
conservation	2019	0.6924	2019	2019	▃▂▂▂
social distancing	2019	0.6924	2019	2019	▃▂▂▂
influenza	2019	17.4734	2020	2020	▂▃▂▂
functional receptor	2019	21.4603	2020	2020	▂▃▂▂
pneumonia	2019	19.8747	2020	2020	▂▃▂▂
sars-cov	2019	19.0675	2019	2020	▂▃▂▂
pathogenesis	2019	19.4661	2020	2020	▂▃▂▂
molecular docking	2019	7.2719	2021	2022	▂▂▃▃
messenger rna	2019	7.3488	2021	2022	▂▂▃▃

#### Analysis of cited references

**Figure 9:**
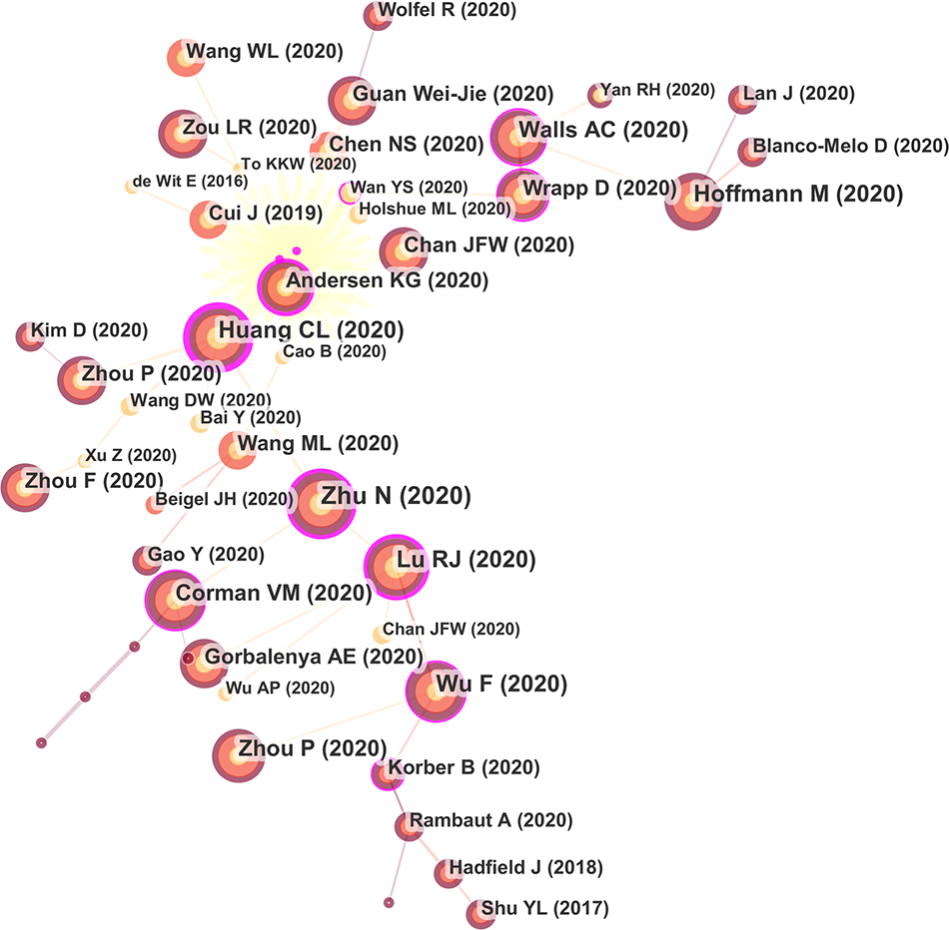
Map of cited references related to COVID-19 genomics.

According to the number of citations, Zhu N is in first place with 1395 citations. The second place is Hoffmann M with 1193 citations. The third place is Huang CL with 1193 citations. The 4^th^ place is Wu F with 974 citations. The 5^th^ place is Lu RJ with 961 citations. The 6th place is Corman VM, with 917 citations. The 7th place is Zhou P with 808 citations. The 8th place is Walls AC with 678 citations. The 9th place is Wrapp D with 649 citations. The 10th place is Gorbalenya AE with 620 citations (**Figure 9**, **Table 6**). Among the 9133 co-cited references retrieved, **Table 6** shows the 43 most frequently cited references, of which A New Coronavirus from Patients with Pneumonia in China, 2019 is the most frequently cited (1395). The majority of the co-cited literature was cited frequently since 2019, which implies that studies related to covid-19 genomics may continue to be a hot spot for future research.

**Table 6:** Centrality and frequency of cited references related to COVID-19 genomics.

Frequency	Centrility	Year	Reference
1395	0.39	2020	Zhu N, 2020, NEW ENGL J MED, V382, P727, DOI 10.1056/NEJMoa2001017
1193	0.05	2020	Hoffmann M, 2020, CELL, V181, P271,DOI 10.1016/j.cell.2020.02.052
1193	0.53	2020	Huang CL, 2020, LANCET, V395, P497, DOI 10.1016/S0140-6736(20)30183-5
974	0.16	2020	Wu F, 2020, NATURE, V579, P265, DOI 10.1038/s41586-020-2008-3
961	0.26	2020	Lu RJ, 2020, LANCET, V395, P565, DOI 10.1016/S0140-6736(20)30251-8
917	0.11	2020	Corman VM, 2020, EUROSURVEILLANCE, V25, P23, DOI 10.2807/1560-7917.ES.2020.25.3.2000045
808	0.00	2020	Zhou P, 2020, NATURE, V588, P0
678	0.1	2020	Walls AC, 2020, CELL, V181, P281
649	0.13	2020	Wrapp D, 2020, SCIENCE, V367, P1260
620	0.00	2020	Gorbalenya AE, 2020, NAT MICROBIOL, V5, P536, DOI 10.1038/s41564-020-0695-z
591	0.05	2020	Chen NS, 2020, LANCET, V395, P507, DOI 10.1016/S0140-6736(20)30211-7
576	0.00	2020	Zhou F, 2020, LANCET, V395, P1054, DOI 10.1016/S0140-6736(20)30566-3
574	0.03	2020	Zhou P, 2020, NATURE, V579, P270, DOI 10.1038/s41586-020-2012-7
529	0.03	2020	Guan Wei-Jie, 2020, N ENGL J MED, V382, P1708, DOI 10.1056/NEJMoa2002032
490	0.32	2020	Andersen KG, 2020, NAT MED, V26, P450, DOI 10.1038/s41591-020-0820-9
482	0.05	2020	Wang ML, 2020, CELL RES, V30, P269, DOI 10.1038/s41422-020-0282-0
478	0.00	2020	Chan JFW, 2020, LANCET, V395, P514, DOI 10.1016/S0140-6736(20)30154-9
440	0.03	2019	Cui J, 2019, NAT REV MICROBIOL, V17, P181, DOI 10.1038/s41579-018-0118-9
440	0.11	2020	Korber B, 2020, CELL, V182, P812, DOI 10.1016/j.cell.2020.06.043
439	0.00	2020	Zou LR, 2020, NEW ENGL J MED, V382, P1177, DOI 10.1056/NEJMc200173
426	0.00	2020	Wang WL, 2020, JAMA-J AM MED ASSOC, V323, P1843, DOI 10.1001/jama.2020.3786
383	0.00	2020	Polack FP, 2020, NEW ENGL J MED, V383, P2603, DOI 10.1056/NEJMoa2034577
335	0.08	2020	Rambaut A, 2020, NAT MICROBIOL, V5, P1403, DOI 10.1038/s41564-020-0770-5
312	0.00	2020	Blanco-Melo D, 2020, CELL, V181, P1036, DOI 10.1016/j.cell.2020.04.026
288	0.03	2018	Hadfield J, 2018, BIOINFORMATICS, V34, P4121, DOI 10.1093/bioinformatics/bty407
264	0.00	2017	Shu YL, 2017, EUROSURVEILLANCE, V22, P2, DOI 10.2807/1560-7917.ES.2017.22.13.30494
262	0.00	2020	Lan J, 2020, NATURE, V581, P215, DOI 10.1038/s41586-020-2180-5
258	0.00	2020	CELL, V181, P914, DOI 10.1016/j.cell.2020.04.011
254	0.00	2020	Wolfel R, 2020, NATURE, V588, P0, DOI 10.1038/s41586-020-2984-3
252	0.00	2020	Gao Y, 2020, SCIENCE, V368, P779, DOI 10.1126/science.abb7498
209 ,	0.00	2020	Beigel JH, 2020, NEW ENGL J MEDV383, P1813, DOI 10.1056/NEJMoa2007764
195	0.05	2020	Wang DW, 2020, JAMA-J AM MED ASSOC, V323, P1061, DOI 10.1001/jama.2020.1585
179	0.00	2020	Bai Y, 2020, JAMA-J AM MED ASSOC, V323, P1406, DOI 10.1001/jama.2020.2565
172	0.00	2020	Holshue ML, 2020, NEW ENGL J MED, V382, P929, DOI 10.1056/NEJMoa2001191
168	0.00	2020	Yan RH, 2020, SCIENCE, V367, P1444, DOI 10.1126/science.abb2762
156	0.00	2020	Chan JFW, 2020, EMERG MICROBES INFEC, V9, P221, DOI 10.1080/22221751.2020.1719902
154	0.16	2020	Wan YS, 2020, J VIROL, V94, P0, DOI 10.1128/JVI.02015-19
142	0.03	2020	Xu Z, 2020, LANCET RESP MED, V8, P420, DOI 10.1016/S2213-2600(20)30076-X
139	0.08	2020	Cao B, 2020, NEW ENGL J MED, V382, P1787, DOI 10.1056/NEJMoa2001282
136	0.00	2020	Wu ZY, 2020, JAMA-J AM MED ASSOC, V323, P1239, DOI 10.1001/jama.2020.2648
126	0.00	2020	Wu AP, 2020, CELL HOST MICROBE, V27, P325, DOI 10.1016/j.chom.2020.02.001
124	0.05	2020	To KKW, 2020, LANCET INFECT DIS, V20, P565, DOI 10.1016/S1473-3099(20)30196-1
123	0.00	2016	de Wit E, 2016, NAT REV MICROBIOL, V14, P523, DOI 10.1038/nrmicro.2016.81

## Discussion

The literature data collected in the manuscript is from the WOSCC, while CiteSpace software uses information from WOSCC and other database networks (Gao et al., 2019). At present, the commonly used bibliometric software includes VOSViewer, CiteSpace, SCI2, NetDraw and HistCite (Shi et al., 2022). CiteSpace, a bibliometric tool developed by Professor Chen Chaomei, is a popular information visualization method in the field of knowledge mapping (Chen et al., 2012). It is good at exploring cooperation, key points, internal structure, potential trends and dynamics in a certain field (Yu et al., 2020; Zhang et al., 2021). It can be seen that bibliometric software has its unique advantages, and citespace software is relatively complete. Therefore, we use CiteSpace (version 5.6. R3) to analyze and visualize the countries, institutions, authors, journals, co-citations, keywords, etc.

The results of the annual publications in this study show a significant overall upward trend in COVID-19. 2019 is the outbreak period of new coronary pneumonia with a relatively small number of publications; 2020 will see 2431 publications, which is in a period of rapid growth; 5852 papers will be published in 2021, which will continue to grow significantly; and 848 papers will be published before March 2022. The upward trend in the number of articles indicates that the genomics research of COVID-19 has become one of the hottest research fields in the past 3 years. The United States and China are countries with a large number of publications, but the level of cooperation between countries is not high. The research institutions are mainly American and Chinese, and the institution with the most publications is the Chinese Acad Sci.

YANG LIU is the author with the largest number of papers and has conducted in-depth research on COVID-19 involving the inactivated SARS-CoV-2 vaccine BBIBP-CorV (Sinopharm), which is a Chinese vaccine approved for entry by TGA and was found to be tolerable and immunogenic in healthy people through a randomized, double-blind, placebo-controlled Phase 1/2 clinical trial (Xia et al., 2021). Their team established a China COVID-19 Single Cell Consortium (SC4), and large RNA-seq datasets covering different disease severities and stages revealed multiple immune signatures of COVID-19 (Ren et al., 2021). D614G in the USA-WA1/2020 strain enhances replication in human lung epithelial cells and primary airway tissue by enhancing viral particle infectivity and D614G mutation enhances viral load in the upper respiratory tract of COVID-19 patients and may increase transmission. Peter Libby is the most cited author. In addition, Christian Weber is the author of numerous publications and citations (Plante et al., 2021). Zhou P is the most cited author and summarizes the basic biology of SARS-CoV-2, including genetic features, potential zoonotic origin, and its receptor binding and clinical and epidemiological features, diagnosis, and countermeasures of COVID-19 (Hu et al., 2021). They also introduce the protein structure of SARS-CoV-2 and structure-based therapeutic development, including antibodies, antiviral compounds, and vaccines, and point out the limitations and prospects of SARS-CoV-2 research (Wang et al., 2020). In the three years after the outbreak of new coronary heart disease, Chinese people made the greatest contribution to the genomics research of new coronary heart disease, focusing on the research and development of a new coronary heart disease vaccine.

The top 10 most cited journals have high impact factors, with NEW ENGL J MED having the highest impact factor of 90.333, indicating that research in the field of COVID-19 genomics has a high international impact and that the concentration of disciplines of COVID-19 genomics-related journals has shifted from molecular, biology, immunology, medicine, pharmacy, clinical and other disciplines to molecular, biology, genetics and other disciplines.

The results of the analysis of keywords show that the research hotspots in the field of COVID-19. Genomics mainly focus on covid 19, sars-cov-2, protein, RNA, angiotensin-converting enzyme II (ACE2), replication, vaccine, expression, spike protein, transmission and so on. Originally dedicated to determining the sequence of nucleotides on a given stretch of DNA, “genomics” has rapidly expanded to a higher level of function-the study of expression profiles and the roles of genes and proteins (Del and Cattaneo, 2012). Remdesivir is currently approved by the FDA for usage in hospitalized COVID-19 patients, although additional information is still required to comprehend the drug’s function in severe COVID-19. Numerous randomized trials of other candidate therapies are underway, including antivirals, antibodies, and immunomodulators (Berlin et al., 2020; Choy et al., 2020; Liu et al., 2020; Wang et al., 2020). Genomic studies into COVID-19 are rapidly increasing. With regard to the S gene and receptor binding domain (RBD), the COVID-19 genome and phylogeny are comparable to those of the SARS-CoV-2, indicating the potential for direct human-to-human transmission (Zhu et al., 2020). SARS-CoV-2 is currently spreading globally as a highly mutated Omicron variant virus. The genomes, dispersal, and effectiveness of vaccinations against Omicron variations are the main topics of the current study (Araf et al., 2022). However, there is limited information on the status of Omicron variants. ANG proposes that SARS-CoV-2 can be divided into two main lineages (L and S), which can be well defined by two tightly linked single nucleotide polymorphisms (SNPs) at positions 8,782 (orf1ab: T8517C, synonym) and 28, 144 (ORF8: C251T, S84L). Among them, the function of the S84L AA change in ORF8 and the possible roles of these two mutations in the pathogenesis of SARS-CoV-2 (Tang et al., 2020).

Since the genetic information of SARS-CoV-2 was first published, various molecular approaches including reverse transcription polymerase chain reaction (RT-PCR), reverse transcription loop-mediated isothermal amplification (RT-LAMP), real-time RT-LAMP, recombinase polymerase amplification (RPA), genome-wide high-throughput sequencing, and clustered regularly interspersed short palindromic repeats/CRISPR-associated system (CRISPR/Cas)-based methods have been used for genetic testing (Rahimi et al., 2021). The use of small non-coding RNAs (either endogenous or exogenous) in RNA interference (RNAi) to match target mRNAs (messenger RNAs) in a sequence-specific manner and silence their expression may potentially combat SARS-CoV-2 (Kim et al., 2009). COVID-19 vaccines based on novel mRNA technology are one of the primary preventive measures to protect humans worldwide from COVID-19 (Laine et al., 2021a; Laine et al., 2021b).

The keyword with the strongest emergent intensity in this study and lasting into 2019 is wuhan. In addition, studies related to functional receptors, influenza, and pathogenesis are also at the forefront of research on COVID-19. In late December 2019, some patients were admitted to the hospital with an initial diagnosis of pneumonia of unknown etiology, the first appearance of which may be considered epidemiologically related to the seafood and wet animal wholesale market in Wuhan, Hubei Province, China (Bogoch et al., 2020; Lu et al., 2020; Rothan and Byrareddy, 2020). Human angiotensin-converting enzyme II (hACE2) has been identified as a functional receptor for SARS-CoV-2. Sun et al. used CRISPR/Cas9 knock-in technology to generate a mouse model expressing hACE2 and discovered that hACE2 mice maintained high viral loads in the lungs, trachea, and brain following intranasal infection. They also found interstitial pneumonia and elevated cytokines were found in aged hACE2 mice that were infected with SARS-CoV-2 (Sun et al., 2020). While the genomic pathogenesis and process of covid-19 are through spike binding to ACE2, allowing SARS-CoV-2 to enter and infect cells, the spike protein must be triggered by a protease called TMPRSS2 (Hoffmann et al., 2020). After the virus enters the host cell and uncoats, the genome is transcribed and then translated, a process involved in the coordination of continuous and discontinuous RNA synthesis mediated by viral replication (Guo et al., 2020; Mousavizadeh and Ghasemi, 2021).

In addition, we should also pay close attention to the still popular research topics of molecular docking and mRNA that appeared in 2021. Molecular docking is structural molecular biology and computer-aided drug design that can be used to virtually screen large compound libraries, rank results, and propose structural hypotheses about how ligands inhibit targets (Morris and Lim-Wilby, 2008). Molecular docking technology is widely used in COVID-19, such as the use of Huashi Baidu formula (HSBDF) in China to treat patients with severe COVID-19, where baicalein and quercetin were found to be compounds with high affinity for ACE2 through network pharmacology and molecular docking (Tao et al., 2020). In clinical observations and randomized controlled trials, Lianhua Qingwen Capsules were protective against SARS-CoV-2 infection and COVID-19 (Xiao et al., 2020). Molecular docking of Lianhua Qingwen capsules shows the interaction mode between effective molecules and targeted proteins, revealing the active pockets of Serine/threonine-protein kinase AKT (Akt1), namely β-carotene, kaempferol, luteolin, naringenin, quercetin, rhein, forsythoside A, and wogonin, which reduce tissue damage and contribute to the elimination of COVID-19 infection (Li et al., 2020; Xia et al., 2020; Chen et al., 2021). In the past few years, messenger RNA (mRNA) has emerged as a promising class of drugs for the treatment of a variety of diseases (Li et al., 2019). T The BNT162b2 messenger RNA vaccine has demonstrated efficacy against SARS-CoV-2 (Bergwerk et al., 2021; Jain et al., 2021).

Most of the current research on COVID-19 has focused on detection techniques, vaccine prevention, and treatment strategies. We found that a few months after the outbreak of COVID-19, Maryam Okhovati et al. found through visual analysis of bibliometrics that the research cluster in China was “quantitative testing” and the United States was “biological assessment” (Okhovati and Arshadi, 2021). There are also the bibliometric statistics of COVID-19 from the perspectives of the eyes (Sánchez-Tena et al., 2021) and ENT department (Wang et al., 2022). We performed statistical and visual analysis of COVID-19 genomics by searching the 2019-2022, countries, institutions, authors, journals, coreferences and keywords. The research hotspots of COVID-19 revolve around quarantine, biological management, angiotensin-converting enzyme-2, RNA-dependent RNA, polymerase, etc. Research fronts and trends focus on molecular docking, messenger RNA, functional receptor, etc. In addition, the existing review articles do not reveal the key issues of the current research hotspots in COVID-19 genomics.

This study has several unique advantages. First of all, we provide the first systematic analysis of genomics studies of COVID-19 through bibliometrics, providing a comprehensive guide for scholars who pay attention to related research. Secondly, the bibliometric analysis provides a more comprehensive insight into hotspots and frontiers than traditional reviews. Last but not least, previous studies have focused on detection techniques used to diagnose COVID-19 infections, emerging variants, and vaccine development and prevention. In the present study, we focused on the role of genomics in COVID-19 and gave a new interpretation of COVID-19 at the molecular level.

Of course, this study also has some shortcomings. First of all, the data for this study were only from the WoSCC database. Other databases such as Scopus or Google were ignored, and some relevant research may be omitted. Secondly, we only used the CiteSpace measurement tool in our survey. VOSviewer (Luo and Lin, 2021), NetDraw (Ramin et al., 2016), HistSite (Ramin et al., 2016) were not involved in the survey. We believed that the combination of one or two databases will make the experimental data more accurate and comprehensive in future studies.

## Conclusion

In summary, this study used CiteSpace software to visualize countries, institutions, authors, journals, literature, keywords, etc. in the WOS from 2019 to 2022 on COVID-19 genomics. Based on the above bibliometric analysis, the main conclusions of this study can be summarized as follows: (1) COVID-19 broke out in 2019, and there was little research on genomics. The number of research in this field has increased rapidly since 2020; (2) The countries, institutions, and authors of COVID-19 genomics are mainly concentrated in countries such as the United States, China, and India; (3)Its research hotspots are focused on the sars-cov-2 virus, functional receptor, pathogenesis, molecular docking, mRNA, which are the prospects for further research in the field of COVID-19 genomics. This research provides a reference for new drug development and vaccine update.

## Data availability statement

The datasets presented in this study can be found in online repositories. The names of the repository/repositories and accession number(s) can be found in the article/supplementary material.

## Author contributions

Conceptualization: Z-YP, XZ. Data curation: X-MP, J-JS. Methodology: X-MP, B-CZ. Visualization: X-MP. All authors contributed to the article and approved the submitted version.
